# Parallel and convergent evolution in genes underlying seasonal migration

**DOI:** 10.1093/evlett/qrae064

**Published:** 2024-11-30

**Authors:** Luz E Zamudio-Beltrán, Christen M Bossu, Alfredo A Bueno-Hernández, Peter O Dunn, Nicholas D Sly, Christine Rayne, Eric C Anderson, Blanca E Hernández-Baños, Kristen C Ruegg

**Affiliations:** Facultad de Estudios Superiores Zaragoza, UNAM, Mexico City, Mexico; Facultad de Ciencias, UNAM, Mexico City, Mexico; Department of Biology, Colorado State University, Fort Collins, CO, United States; Facultad de Estudios Superiores Zaragoza, UNAM, Mexico City, Mexico; Department of Biological Sciences, University of Wisconsin-Milwaukee, Milwaukee, WI, United States; Department of Biological Sciences, University of Wisconsin-Milwaukee, Milwaukee, WI, United States; Department of Biology, Colorado State University, Fort Collins, CO, United States; Department of Biology, Colorado State University, Fort Collins, CO, United States; Facultad de Ciencias, UNAM, Mexico City, Mexico; Department of Biology, Colorado State University, Fort Collins, CO, United States

**Keywords:** parallel evolution, convergent evolution, *Geothlypis trichas*, candidate genes, genomic variation, migration

## Abstract

Seasonal migration has fascinated scientists and natural historians for centuries. While the genetic basis of migration has been widely studied across different taxa, there is little consensus regarding which genomic regions play a role in the ability to migrate and whether they are similar across species. Here, we examine the genetic basis of intraspecific variation within and between distinct migratory phenotypes in a songbird. We focus on the Common Yellowthroat (*Geothlypis trichas*) as a model system because the polyphyletic origin of eastern and western clades across North America provides a strong framework for understanding the extent to which there has been parallel or convergent evolution in the genes associated with migratory behavior. First, we investigate genome-wide population genetic structure in the Common Yellowthroat in 196 individuals collected from 22 locations across breeding range. Then, to identify candidate genes involved in seasonal migration, we identify signals of putative selection in replicate comparisons between resident and migratory phenotypes within and between eastern and western clades. Overall, we find wide-spread support for parallel evolution at the genic level, particularly in genes that mediate biological timekeeping. However, we find little evidence of parallelism at the individual SNP level, supporting the idea that there are multiple genetic pathways involved in the modulation of migration.

## Introduction

Seasonal migration, which involves yearly movements between breeding and wintering grounds, is a widespread phenomenon in the animal kingdom (Adriaensen & Dhondt, 1990; Liedvogel & Delmore, 2018). Rather than simply being gained or lost, migratory behavior is generally thought of as a highly polygenic trait that evolves under a threshold model of evolution (Pulido, 2011). Genetic variation controlling to the propensity to migrate is thought to be maintained within populations and, as such, can be rapidly acted upon by selection (Berthold & Pulido, 1994). As a result, populations can repeatedly switch between migratory and resident behaviors, as observed across the evolutionary history of various species (Able & Belthoff, 1998; Liedvogel & Delmore, 2018; Pulido, 2007), similar to the repeated loss and gain of eye function in several species of cave dwelling fishes or body armor in stickleback fish (Colosimo et al., 2005; Jones et al., 2012; Sifuentes-Romero et al., 2023). In migratory birds, for example, captive breeding experiments on black-capped warblers have shown that under the right environmental conditions a population can shift from completely migratory to completely resident in a few short generations (Pulido & Berthold, 2010; Pulido et al., 1996). Comparing populations at the extreme ends of the spectrum of migratory behavior (i.e., resident versus fully migratory) using recent advances in genomics provides a unique opportunity to identify the genetic polymorphism underlying this complex trait and how genes associated with migration are modulated within and across populations, rather than complete gains or losses of migratory behavior.

Current migration genetics research has revealed a lack of consensus regarding the genes that may play a role in bird migration (Bazzi et al., 2017; Lugo Ramos et al., 2017). For instance, recent research in Willow Warblers identified two genes putatively underlying migratory direction between two of the European subspecies (Sokolovskis et al., 2023), but these same genes did not account for directional differences in a closely related Siberian form (Lundberg et al., 2017). Another study found clock-linked genes regulate migratory timing in American kestrels (Bossu et al., 2022), but these genes largely differed from those identified in migratory timing studies in several other species (Johnsen et al., 2007; Liedvogel et al., 2009; Saino et al., 2015). Similarly, genes related to fat deposition and aerobic capacity in migrant and resident European blackbirds (Franchini et al., 2017) were not the same as the genes found to be associated with lipid metabolism in Gray Catbirds (Corder et al., 2016). To better understand the genetic basis underlying this dynamic phenotype, it would be valuable to compare signatures of selection in closely related groups that have different migratory behaviors (e.g., Fudickar et al., 2016).

Genetic convergence occurs when different genetic changes result in the same phenotypes (Arendt & Reznick, 2008), while parallel evolution occurs when the same genetic changes result in similar phenotypes (Bailey et al., 2017). At present, there is no clear consensus as to how widespread convergent and parallel evolution are in nature because it is often difficult to distinguish between the two (Pickersgill, 2018; Stern, 2013). Studies examining replicated transects of hybrid zones in various taxa, such as European crows, Bahama mosquitofish, and three-spine stickleback, indicate that both genetic convergence and parallel evolution are frequently observed mechanisms of evolution (Langerhans, 2018; Marques et al., 2022; Vijay et al., 2016). These studies offer valuable insights into the genetic basis of key fitness-linked traits, including bill size, body shape, feather coloration, aggression, armor plating, and spine length, as well as the extent to which they have evolved through convergence or parallel genetic mechanisms. This existing research demonstrates that the prevalence of genetic convergence versus parallel evolution can differ depending on the phenotypes, traits, and species under investigation. Therefore, it is necessary to examine each phenotype on a case-by-case basis.

Here we study replicate comparisons between migratory and resident forms of the Common Yellowthroat (*Geothlypis trichas*) to quantify the degree of genetic convergence or parallelism in genes linked to migratory behavior. This species is an ideal system in which to address these questions because migratory and resident populations within the eastern and western groups are known to have distinct evolutionary origins (Ball & Avise, 1992; Escalante et al., 2009). Phylogenetic analyses have shown that *G. trichas* is a polyphyletic species where the eastern group is more closely related to two Mexican resident species: *G. nelsoni* and *G. flavovelata*, and the western group is more closely related to *G. beldingi*, a resident species found in Baja California Sur, Mexico (Escalante et al., 2009). Overall, our study has three main goals: (a) to characterize genome-wide population genetic structure across the breeding range, (b) to identify candidate genes involved in migratory behavior across three replicate comparisons of migratory and resident populations that span multiple environments and latitudes, and (c) to quantify the extent of parallel versus convergent evolution in genes linked to migratory behavior within the Common Yellowthroat compared to other migratory bird species. By having three different comparisons between migrant and resident phenotypes that span multiple distinct evolutionary histories, different environments and latitudes—southeast versus migrant, southwest resident versus migrant, and northern California coastal resident versus migrant—we are able to control for multiple confounding variables and examine how genetic convergence and parallel evolution has contributed to the modulation of migration across distinct evolutionary lineages.

## Methods

### Sampling and DNA extraction

**Figure 1.**
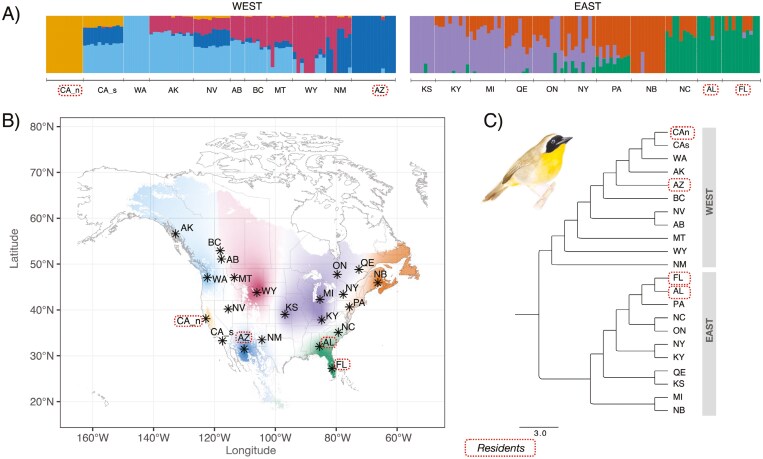
Population structure results obtained with AdmixPipe performed for each main group: West and East (A). Geographic distribution of population structure (B). Evolutionary relationships inferred with Treemix (C). The letter codes represent different sampled localities (see Supplementary Table S1), and different colors represent main clusters found. Resident populations are represented inside dotted circles.

We compiled a collection of 202 blood and feather samples from 22 locations across the breeding range of the Common Yellowthroat, *Geothlypis trichas* (Figure 1, Supplementary Table S1). While 18 populations were migratory, we specifically included sampling of two resident populations in the West (northern California and Arizona) and two resident populations in the East (Florida and Alabama) to allow replicated migratory-resident comparisons. Not only are these phylogenetically independent resident populations, they span multiple environments and latitudes-southeast resident versus migrant, southwest resident versus migrant, and northern California coastal resident versus migrant, which allows us to tease apart different selection pressures and latitudes. DNA was extracted from all samples using the Qiagen DNeasy Blood and Tissue extraction kits (Qiagen Inc., Valencia, CA, USA). For blood samples, we followed the standard tissue extraction protocol using 20 µl of blood suspended in Queen’s lysis buffer (Seutin et al., 1991). For feathers, at least one calamus/sample was cut using a sterile razor and incubated during the tissue digestion step for 24 h with the addition of 10 µl of dithiothreitol (DTT). We conducted the elution step twice to obtain final volumes of 120 µl of purified DNA. DNA quantification was done using a Qubit dsDNA HS Assay kit (Thermo Fisher Scientific).

### Low coverage whole genome re-sequencing

We used a modified version of Illumina’s Nextera Library Preparation protocol to prepare whole-genome sequencing libraries and pooled the libraries by equal mass prior to sequencing. The first step in library prep was the tagmentation reaction that fragmented DNA and we then tagged the DNA with adapter sequences. Library amplification was completed using a limited-cycle PCR program, followed by a reconditioning PCR step, and a cleaning step with AMPure XP beads that size selects short library fragments. We quantified the library with a Qubit plate reader and normalized the quantity of libraries to be pooled together. Final libraries with a volume of at least 20 µl and a concentration of at least 2 ng/µl were sequenced on two Illumina HiSeq4000 (Illumina) lanes and on one Illumina Novaseq 6000 lane by Novogene Corporation (en.novogene.com).

### Processing raw reads and variant detection

A pipeline adapted from the Genome Analysis Toolkit (GATK) Best Practices Guide (Van der Auwera et al., 2013) was used to process raw reads before genotype calling. Briefly, we trimmed adapters using TrimGalore (Krueger, 2020), marked PCR duplicates using *samtools* (Li et al., 2009), and paired-end raw sequence reads were aligned to the Common Yellowthroat reference genome using *bwa-mem* (Li & Durbin, 2009). Read groups (sample, lane, library) were added using *picard* (http://broadinstitute.githut.io/picard), and bam files were merged using *samtools* (Li et al., 2009) if individuals were sequenced on multiple lanes. Genotype calling was performed with GATK HaplotypeCaller (Van der Auwera & O’Connor, 2020) and genotypes were filtered for minor allele frequency (maf = 0.05 and max-maf = 0.95), quality (*q* = 30), and allelic number (min-alleles = 2, max-alleles = 2) using *vcftools* v.0.1.16 (Danecek et al., 2011). The dataset was further filtered for missingness, keeping only variants identified in 75% of the individuals. Finally, missing genotypes were imputed using beagle 4.1 (Browning & Browning, 2007). For downstream analyses that used genotyped probabilities, we calculated genotype probabilities using ANGSD v.0.921 (Korneliussen et al., 2014).

### Genome annotation

To annotate the Common Yellowthroat genome (Bobowski et al. in review), we used the best practices of the *maker* pipeline (Cantarel et al., 2008). Repeat masking was carried out using Repeatmasker (Smit et al., 2013), while SNAP (Korf, 2004) and AUGUSTUS (Stanke et al., 2006) were used as *ab initio* gene predictors. We utilized the gene predictions of the chicken in AUGUSTUS and proteins of the Zebra finch (*Taeniopygia guttata*) from Swiss-Prot (Bateman, 2019) and verified gene models from Zebra finch (taeGut-3.2.4) to support the gene prediction.

### Population structure

Previous research suggests that a deep divergence between eastern and western forms of the Common Yellowthroat exists as a result of their polyphyletic origins (e.g., Escalante et al., 2009). To confirm the existence of a deep divergence within the species using genome-wide data, we utilized Treemix v1.1 to reconstruct the evolutionary relationships among the 22 populations sampled across their breeding range (Pickrell & Pritchard, 2012). This program uses genome-wide allele frequency data to infer evolutionary relationships and potential migration events. We then used the Popgen Pipeline Platform (PPP) (Webb et al., 2021) to convert our imputed vcf file to the treemix input file using the *vcf_to_treemix.py* function. When running Treemix, we generated a bootstrap replicate by resampling blocks of 500 SNPs.

To assess population genetic structure within eastern and western clades we conducted both a Principal Component Analysis (PCA) and ran ADMIXTURE (Alexander et al., 2009). To conduct the PCA we used the single read sampling method (srs) in the R package srsStuff (eriqande.github.io). Single read sampling randomly samples one read per site, which eliminates coverage issues that can mask PCA patterns. We exported allele depths using *bcftools* (Danecek et al., 2021). To better assess relatedness and geographic variation within and between eastern and western clades, we created PCAs for all the data, as well as for the East and West separately. Because the presence of related individuals can obscure geographic patterns, we also used the srsStuff R package to identify related individuals based on pairs or clusters of individuals that exhibit high genetic covariance. For the six pairs with genetic covariances ⋝0.3, we kept one individual per pair and filtered out the remaining six individuals.

To parse and filter imputed VCF files in preparation for ADMIXTURE (Alexander et al., 2009), we used AdmixPipe v3 (Mussmann et al., 2020). East and West variant datasets were filtered based on a minimum allele frequency of 0.05 and thinned to include one locus within 1,000 base pairs to remove linked loci. ADMIXTURE was run on the resulting filtered Eastern and Western datasets at *K* values from one to six, with five independent replicates for each *K* value. Admixture runs were considered complete when log-likelihood converged and increased by less than 10^−4^ between iterations. Values of cross validation error were plotted to visualize the best *K* values. Additionally, we measured the nucleotide diversity (π) per window using variant + invariant sites with no MAF filter and obtained mean values in vcftools v.0.1.16 (Danecek et al., 2011) (--window-pi in 10 Kb window) to evaluate genetic diversity variation within populations.

### F_ST_ outlier detection

We used ANGSD v.0.921 (Korneliussen et al., 2014) to generate site allele frequency files for each of three resident populations-northern California (CA_n), Arizona (AZ), and Florida and Alabama combined (East). To create the site allele frequency files for the East and West migratory populations, we merged data between sampling sites that exhibited no signs of population structure. In the West, we combined individuals from the Alberta (AB), Alaska (AK), British Columbia (BC), southern California (CA_s), Montana (MT), New Mexico (NM), Nevada (NV), Washington (WA), and Wyoming (WY) migratory populations. In the East, we combined individuals from migratory populations in Kansas (KS), Kentucky (KY), Michigan (MI), New Brunswick (NB), New York (NY), Ontario (ON), Pennsylvania (PA), and Quebec (QUE). North Carolina (NC) was excluded from the Eastern group because it fell on the border of the eastern resident and migrant populations and was genetically ambiguous (Figure 1, Supplementary Figure S1).

**Figure 2.**
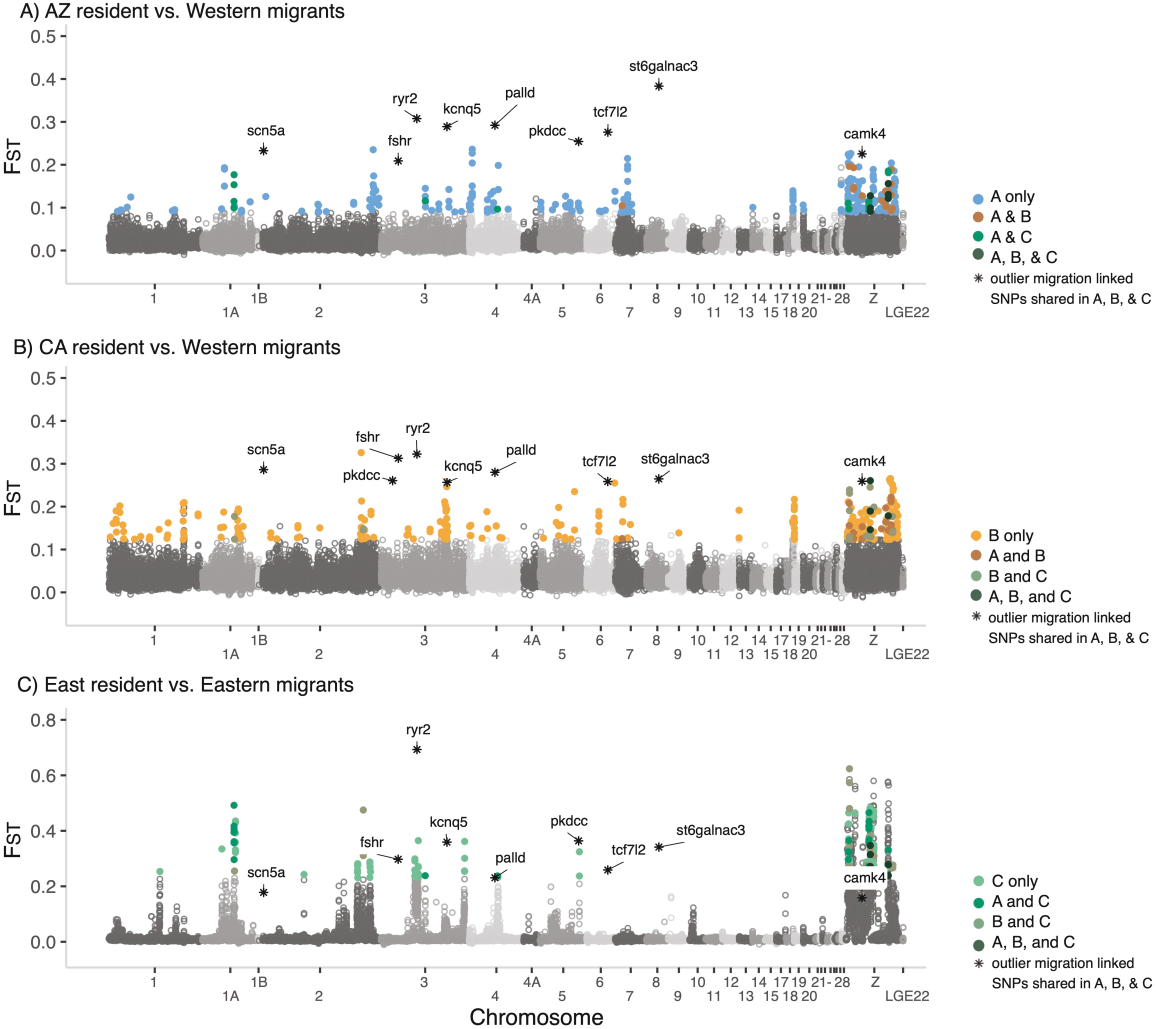
Genetic differentiation, F_ST_, estimated in 50 kb sliding windows across the genome between a set of three resident-migrant comparisons. (A) AZ resident vs. Western migrants, (B) CA resident vs. Western migrants, and (C) East residents vs. Eastern migrants. Genomic regions of extreme differentiation (>99th percentile) identified in a single comparison are highlighted in each panel. Additionally, shared windows between two comparisons (A and B, A and C, and B and C) are highlighted as are windows shared across all three comparisons (A, B, C). The nine outlier migration-linked genes between all three comparisons are highlighted with asterisks. Colors correspond to Figure 4 and Table 1.

To determine global F_ST_ and identify regions potentially significant to migration strategy, we utilized realSFS in ANGSD v.0.921 (Korneliussen et al., 2014). We calculated F_ST_ within 50 kb sliding windows with 25 kb steps, set the max number of sites to 15 million bases (-nSites) and considered outlier regions that fell within the 99th percentile. We estimated F_ST_ between the following three resident-migrant comparisons: (a) Arizona residents vs. West migrants, (b) northern California residents vs. West migrants, and (c) East residents (Florida and Alabama) vs. East migrants. To narrow our F_ST_ scan to variants that were related specifically to migration rather than demographic history and population differentiation, we excluded any variants that were outliers in comparisons between migratory populations in different regions (but within the East or West), as these would represent population differentiation unrelated to migration strategy. For example, in the East this included comparisons of migrants in the Midwest (Michigan) versus migrants along the Atlantic Coast (New Brunswick). In the west, this included migrants in the Pacific Northwest (Washington) versus migrants in the West Central region (Wyoming). We additionally excluded variants identified as outliers between resident population comparisons in the West and East regions (e.g., northern CA vs AZ and FL vs AL, respectively). We used bedtools v.2.30.0 (Quinlan & Hall, 2010) to remove overlapping regions in the East and West separately due to their distinct evolutionary histories. To identify parallel and convergent outlier windows among the three resident-migrant comparisons, we utilized bedtools v.2.30.0–intersect (Quinlan & Hall, 2010) and visualized results in Manhattan plots (Figure 2).

To identify outlier genes specific to migration, we calculated site-wise F_ST_ estimates following the same steps as the outlier window identification methods described above. Loci were considered outliers if they fell within the 99th percentile of resident-migrant comparisons and were also not identified as outliers in migrant-migrant and resident-resident comparisons. We intersected the site-wise F_ST_ outliers with the annotated genome using bedtools --closest (Quinlan & Hall, 2010) and focused on variants that were found in or within 25 kb of genes linked to migration based upon an exhaustive literature search (Table 1).

### Genome-wide selection scan

To identify genome-wide selection signatures between populations with differing migratory phenotypes, we utilized principal component analysis-based selection statistics that have been expanded to consider genotype uncertainty (Meisner et al., 2021). We first used ANGSD (Korneliussen et al., 2014) to generate genotype likelihood *beagle* files for the East and West separately. Subsequently, we employed the PCAngsd framework (Meisner & Albrechtsen, 2018) to perform a genome-wide selection scan, which uses an extended model of FastPCA (Galinsky et al., 2016). This scan incorporates population structure by estimating individual allele frequencies iteratively. We converted the PCAngsd output statistics into *p*-values using a χ^2^ distribution and identified genes that had strong selection signals within 25 kb of the gene (i.e., *p*-values < 0.05). We then determined whether any of the genes with a signal of selection overlapped with migration-linked genes identified in the F_ST_ outlier scan.

Because the above selection scan does not indicate on which population selection is acting, we additionally calculated cross population extended haplotype homozygosity (XP-EHH; Sabeti et al., 2007) to identify recent positive selection in all three comparisons. This test detects selective sweeps in which the selected allele has approached or achieved fixation (above 80%; Sabeti et al., 2007; Voight et al., 2006) in one population (i.e., the resident population), but not all populations (i.e., the migratory populations), or vice versa. As this scan focuses on more recent signals of selection, it will not be influenced by historical demographic processes. The imputed vcf file was first phased with beagle 5.1 (Browning et al., 2018) and then realigned to the zebra finch reference genome assembly (taeGut-3.2.4) in satsuma2 (Grabherr et al., 2010) to create a phased chromosomal and imputed dataset. The phased chromosomal vcf was then subset into each chromosome and for each of the five populations used in the replicate comparisons: AZ resident, northern CA resident, West migrants, East residents, and East migrants. We used selscan v2.0.1 (Szpiech & Hernandez, 2014) to run the XP-EHH test. Given the XP-EHH value can be positive or negative depending on which population selection is acting in, the *p*-value was based on the rank of the absolute value of the XP-EHH statistic. We considered variants to be significantly selected in one population if -log(*p*-value) was greater than two (*p*-value = 0.01). We then annotated the predicted function of these selected variants using SNPEff (Cingolani et al., 2012).

## Results

### Variant detection

We sequenced 202 individuals over three lanes of sequencing. Five of the 202 samples were only sequenced on one lane because their coverage was within our goal (2x), while the remainder were re-sequenced on additional lanes due to low yield of early sequencing runs. Quality of reads was very high across libraries, as indicated by an average Phred quality score of 36. The final dataset included 202 Common Yellowthroat (Supplementary Table S1) with an average coverage of 2.88X.

Processed reads aligned to the reference genome with an average overall rate of 99%. An average of 97% of mapped reads were retained after filtering for multiple hits and were used for downstream analyses. We built a catalog of 74,750,284 loci. After filtering for minor allele frequency (maf = 0.05 and max-maf = 0.95), quality (*q* = 30), allelic number (min-alleles = 2, max-alleles = 2) and missingness (max-missing = 0.75) in *vcftools* v.0.1.16 (Danecek et al., 2011), we obtained a final dataset of 18,130,407 SNPs distributed across the genome. We further removed six individuals due to relatedness identified in srsStuff R package (covariance ⋝ 0.3).

### Population structure

At a broad scale, the analysis of population relationships using Treemix revealed general patterns that confirms that the main separation within Common Yellowthroats is between populations in the East and West (Figure 1C). Additionally, within the East, the resident populations are clustered in one monophyletic group, whereas in the West the two resident populations do not cluster together (Figure 1A). This supports the polyphyletic relationship between eastern and western groups. Overall, the PCA and ADMIXTURE analysis confirmed the Treemix results showing that the primary division is between populations in the East and West (Supplementary Figure S2A). When PCAs were constructed for populations within the East and West separately, resident, and migrant populations grouped separately (Supplementary Figures S2B and S2C). Because the admixture results were inconclusive regarding the best *K* value, we selected the *K* values that represented consistent, meaningful geographic structure identified in the admixture plots (see Supplementary Figure S1). In the West we identified a total of four genetic clusters (Figure 1), including two distinct resident populations in northern California and Arizona, as well as two clusters of migrants in the Pacific Northwest and West Central regions. In the East we identified three genetic clusters. Individuals from the resident populations (AL and FL) clustered together and individuals from NC displayed moderate signs of admixture between the resident populations and northern migratory populations (Figure 1A). In turn, Eastern migrant populations formed two main genetic clusters, with the Northeast and Midwest coming out as separate regions with some admixture present in New York and Quebec. Mean values of nucleotide diversity were similar among populations, where resident populations resulted in PI values of 0.00119 for Arizona, 0.00102 for northern California, and 0.00164 for Florida and Alabama, while migratory populations resulted in PI values of 0.00127 and 0.00172 for western and eastern groups, respectively.

### Candidate loci resulting from F_ST_ outlier and selection scans

**Figure 3.**
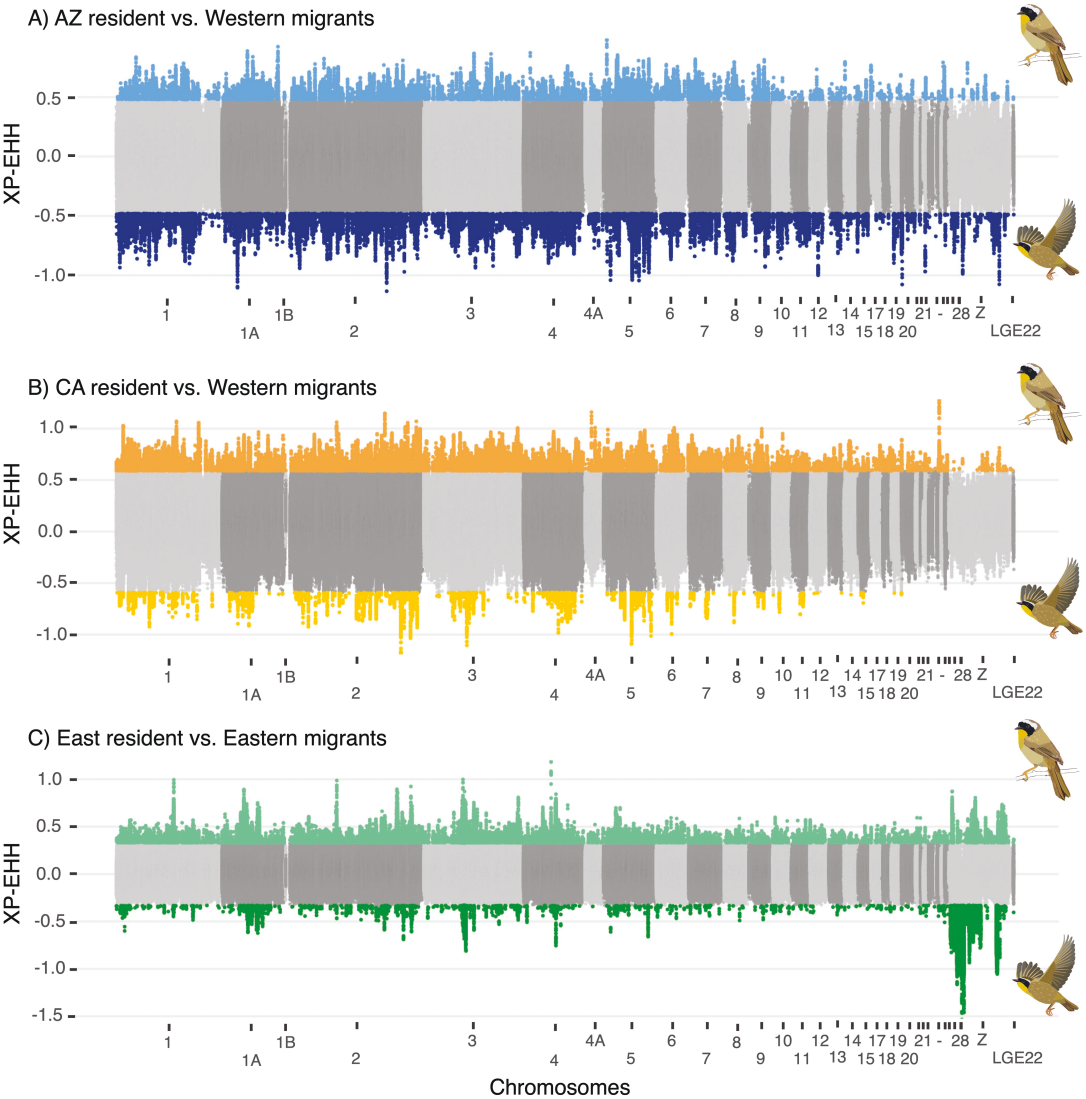
Manhattan plots of the cross population extended haplotype, XP-EHH, candidate loci identified in resident (positive XP-EHH statistic) and migrant (negative XP-EHH statistic) populations for three resident-migrant comparisons: (A) AZ resident vs. Western migrants, (B) northern CA resident vs. Western migrants, and (C) Eastern residents vs. Eastern migrants. Illustrations by Erica Robertson (*Geothlypis trichas*) and used with permission.

Here, we compared results from F_ST_ outlier scans, ANGSD selection scans, and XP-EHH directional selection scans to identify candidate loci underlying migratory phenotypes in three replicate resident-migrant comparisons. Window-based F_ST_ outlier scans and site-based F_ST_ outlier and selection scans showed distinct patterns between the East and West comparisons (see Supplementary Results  *-F*_*ST*_  *outlier scan and Genome-wide selection scan-* for distinct numbers for each comparison; Figure 2, Supplementary Figures S3 and S4); however, by focusing on the union of these analyses, we demonstrated parallelism across the replicate populations on the genic level, not the locus level. Specifically, the site-based F_ST_ outlier analyses identified 86,308 outlier loci (99th percentile) in total, with only 443 shared between replicate comparisons. The ANGSD selection-based scans identified 554,082 loci with a signature of selection (*p*-value < 0.05), while the XP-EHH analyses identified 131,616 loci with a signature of directional selection (Figure 3). Of these selected loci, 4,112 loci were shared between the replicate comparisons for F_ST_ and ANGSD selection scan and 3,001 were shared between the XP-EHH selection scans, respectively.

We subsequently narrowed our focus to loci found in or near (within 25 kb) migration-linked genes that have been identified either in (a) in genome scan studies involving migratory organisms (mostly birds); (b) as being involved in circadian clocks more generally (e.g., identified in rodents or other model organisms); (c) involved in morphology that could pertain to aerodynamics; or (d) important to metabolism across a variety of organisms (Table 1). Directional selection scans corroborated selection footprints in 31 migration-linked genes (Table 1). Moreover, we identified a greater proportion of alleles and genes with a selection signal in the East resident and northern California resident population, a pattern not shared in the Arizona resident population. For instance, in the northern California resident-migrant comparison, we identified 339 selection footprints in loci near migration-linked genes, 314 outliers suggesting that selection happened in the resident population, and only 25 outliers indicating that selection happened in the migratory populations (Supplementary Table S2). Similarly, in the East, a total of 748 outliers were detected, of which 564 suggested selection in the East resident population, and 184 loci suggested selection in the migratory population. In contrast, the southernmost resident-migratory comparison in the West suggests a more equitable, yet differentially skewed, distribution of selection signals. A total of 420 outliers were detected in the Arizona resident-migrant comparison, with 181 suggesting selection in the Arizona resident population and 239 suggesting selection in the western migratory population. While the directionality of the selection footprints differed across comparisons, the distribution of predicted functions of the selected loci was similar. The majority of loci identified in selective sweeps were found in noncoding regions (e.g., intronic, intergenic regions, and regulatory regions; Supplementary Table S2). We identified very few synonymous mutations, one missense mutation, and no nonsynonymous mutations (Supplementary Table S2). This suggests that cis-regulatory elements that regulate the expression of migration-linked genes underlie the differentiation of migratory behavior rather than causal mutations in coding regions.

**Figure 4.**
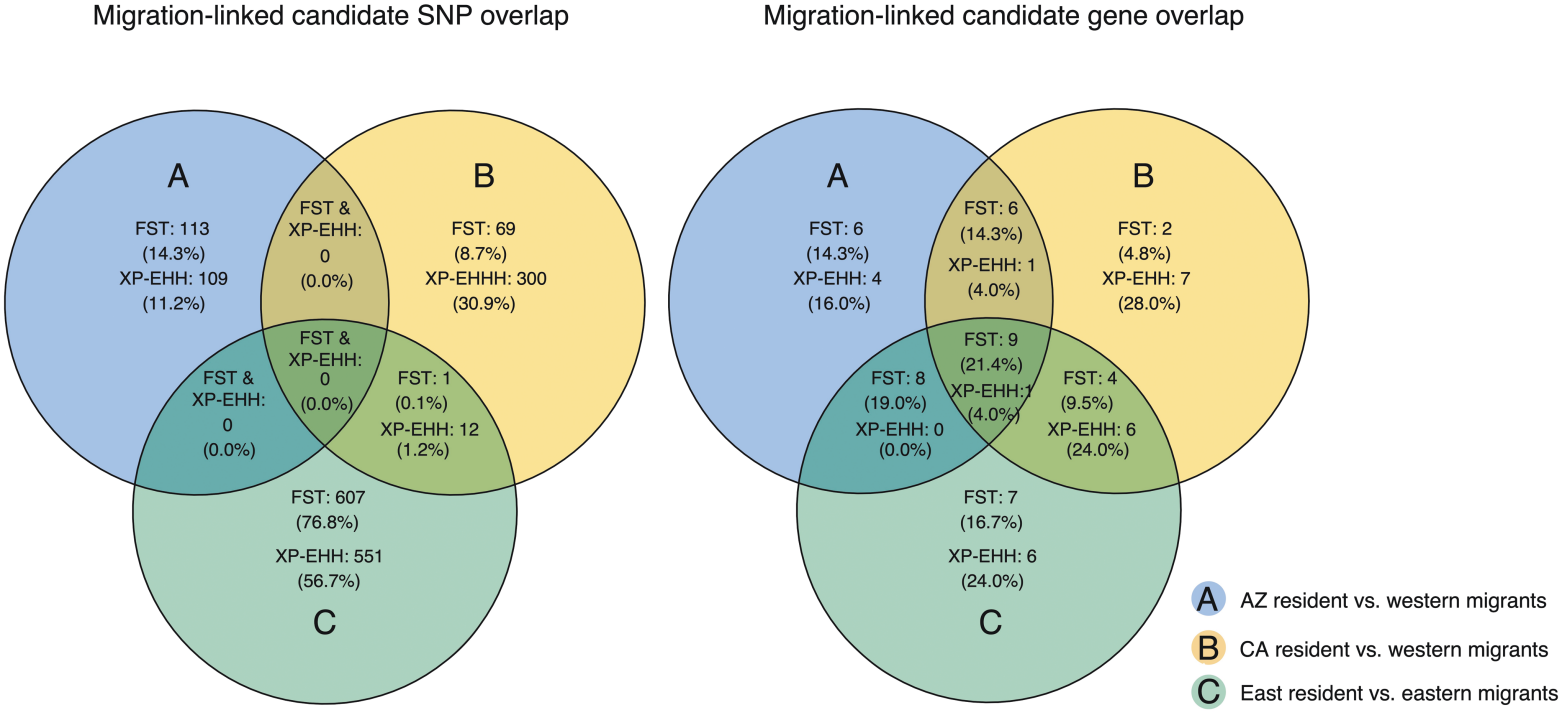
Venn diagrams that express parallelism between three replicate resident-migrant comparisons in migration-linked outlier variants (A) and outlier genes (B) for both the F_ST_ outlier scan and the XP-EHH candidate loci identified in the resident populations.

In Arizona, we identified 114 outlier loci associated with migration-linked genes, 70 outlier loci identified in the California comparison and finally we identified 608 outlier loci in the East resident-migrant comparison. On the SNP-level, there was only one locus shared between the California and East resident-migrant comparison and none were shared among all three comparisons. On the genic level, 29 migration-linked genes were identified in the Arizona comparison, 21 genes were identified in the California comparison, and 28 were identified in the East migrant-resident comparison. Altogether, six genes were shared between the Arizona and California resident-migrant comparisons, eight genes were shared between the AZ and the East resident-migrant comparisons, four genes were shared between CA and East resident-migrant comparisons, and nine genes were shared between all three resident-migrant comparisons (Table 1; Figure 4).

### Genes associated with migration

Using the genes identified in both the F_ST_ outlier and both selection scan approaches, we categorized genes associated with migration into the following three groups (Table 1): (a) migration timing, including circadian rhythms, and metabolic sensors and photoperiodic pathways which regulate clock function, (b) the energetics of flight, including metabolism and energy expenditure, and (c) morphology potentially related to the aerodynamics of flight. Overall, we identified 30 genes linked to migration timing (Table 1), 17 of which are identified as outliers in at least two comparisons and 13 of which were only found in one comparison. Five of the 30 genes were core clock genes underlying circadian rhythms, while 12 genes were known to modulate or entrain circadian rhythms via light input or metabolic sensor pathways, five were clock-controlled genes, six were known to regulate clock, and two were involved in the sleep-wake cycle. Alternatively, we identified six genes associated with the energetics of flight, all except one, ggt7, of which were found in multiple comparisons as well as multiple other bird species (e.g., Franchini et al., 2017; Lundberg et al., 2017). In general, these genes were involved in lipid and glucose metabolism, and migration molt. Finally, we identified six genes associated with morphology, all except one, tnfrsf11a, of which were identified in multiple comparisons and all except one of which was found in other bird species. Many of these were known to be involved in cytoskeleton regulation, bone metabolism, and bone mass.

**Table 1.**
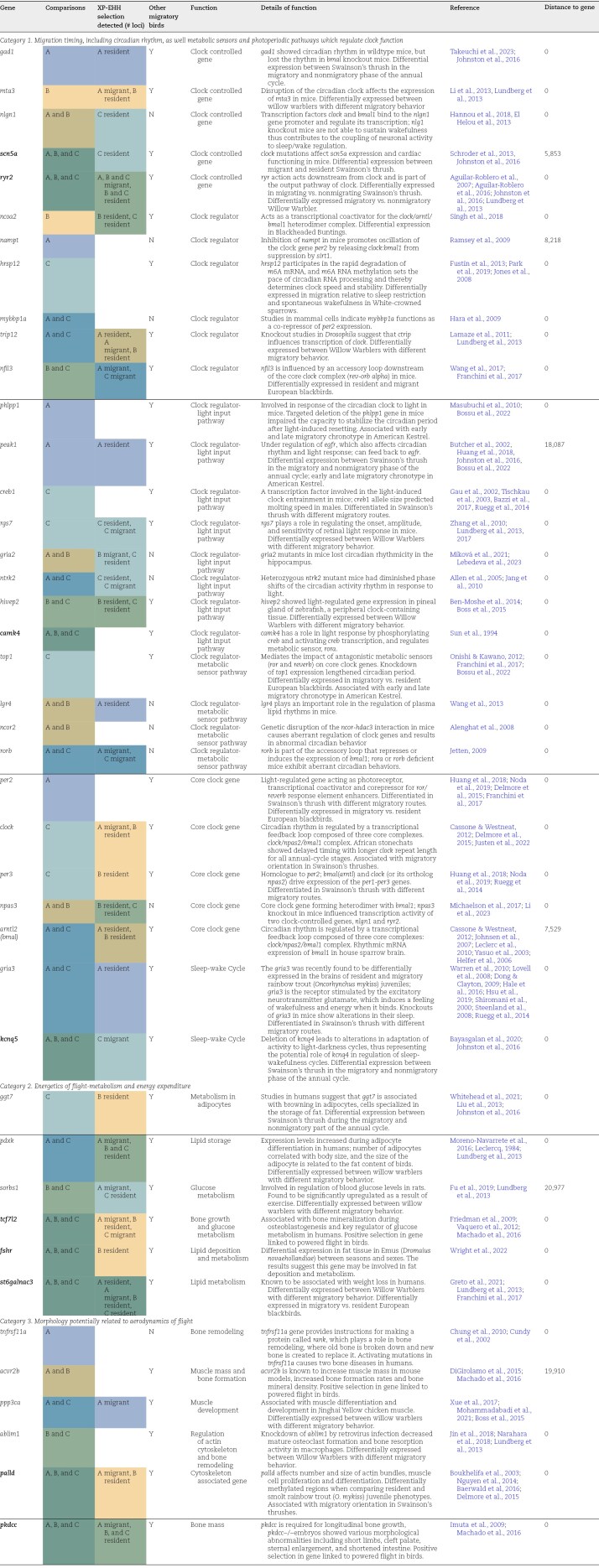
List of migration-linked genes identified by F_ST_ outlier and selection scans as being potentially under selection in pairwise resident-migrant comparisons. Colors correspond to Figure 2 and Figure 4.

A refers to AZ resident vs. West migrant comparison, B refers to the northern California resident vs. West migrant comparison and C refers to the East resident vs. East migrant comparison. The directional selection scan, XP-EHH, identifies which population selection was acting on for each gene. The genes are categorized by putative function in relation to migration as well as by the number of pairwise comparisons in which they were significant. Putative function and references in which function was identified are included. The color of the Comparisons and XP-EHH columns correspond to Venn diagram colors (Figure 4). Specifically, the color of the XP-EHH column corresponded to which resident populations selection was acting in (or which migrant populations selection was acting in, secondarily). Genes in bold are shared between all three resident-migrant comparisons.

## Discussion

Although there have been numerous studies focused on understanding the genetic basis of migratory behavior, none of them have used genome-wide analyses with replicate population comparisons in which different migratory phenotypes exist in closely related groups. Replicated comparisons of migratory and resident populations offer a unique opportunity to observe the degree of genetic convergence or parallelism underlying this notoriously complex phenotype. Here we analyze the population genomic structure of the Common Yellowthroat across the breeding range and find strong population structure between eastern and western groups as well as between resident and migratory forms within each group. When comparing signatures of putative selection between migrants and residents in the west and east, we observed limited evidence of genetic parallelism at the individual SNP level, but strong support for parallel evolution at the genic level. Parallel evolution was strongest in genes linked to morphology and metabolic processes necessary for meeting the physiological demands of migratory flight, as well as genes related to biological clock mechanisms likely involved in migratory timing. Alternatively, genetic convergence was also widespread, with many different migration-linked genes coming out as important in one or two comparisons, but not all three. These findings underscore the existence of multiple evolutionary pathways leading to similar adaptations and help clarify why previous studies have failed to find parallelism in genes associated with migration (Lugo Ramos et al., 2017).

### Geographic structure and evolutionary relationships

The Common Yellowthroat represents a unique system that exhibits strong geographic variation across its entire range. Previous work has identified numerous mechanisms leading to this population structure, including historical demography (Ball & Avise, 1992), adaptation (Bolus, 2014), dispersal capability (Escalante et al., 2009), and sexual selection (Sly et al., 2022). However, much of the previous work was restricted by small sample sizes (~30 individuals) and/or the use of a single molecular marker (e.g., mitochondrial DNA; Escalante et al., 2009). In this study, we used genome-wide sequencing and comprehensive range-wide sampling to clarify the evolutionary relationships within this species. Similar to previous studies, our findings indicate that the primary genetic division exists between the eastern and western clades. Additionally, within the west, we identified genetic breaks between CA residents, AZ residents, and the western migratory populations, with weaker and more clinal variation across the western migratory populations (AK, NV, AB, BC, MT, WY, NM; Figure 1). Moreover, sampled populations within the East clustered in three distinct genetic groups: Northeastern (NB, PA), Midwest (KS, KY, MI, QUE, ON, and NY) and a Southeastern resident cluster (FL and AL), with a transition zone in North Carolina (NC) (see Figure 1A, Supplementary Figure S1). Previous multi-species phylogenetic analysis suggests that the main split between the East and West groups resulted from the Eastern form being sister species to two resident species in northwestern and central Mexico, *Geothlypis nelsoni* and *G. flavovelata*, while the Western form evolved from a common ancestor shared with *G. beldingi,* a resident species in Baja California Sur, Mexico (Escalante et al., 2009). Our results, which identified a strong split between eastern and western groups with weaker structure within each group, lend further support to the hypothesized polyphyletic origins of Common Yellowthroats. Here we take advantage of the polyphyletic origin of the two main clades to assess the extent to which there has been parallel or convergent evolution in the genes associated with migration or residency in the east and the west.

### Selection signals in replicate comparisons

Genomic evidence of selection has been widely viewed as an effective approach for exploring the potential genetic mechanism of phenotype differentiation (Amaral et al., 2011; Andersson & Georges, 2004; Oleksyk et al., 2010). To identify loci underlying differences between resident and migratory phenotypes of the common yellowthroat, we combined F_ST_ outlier scans, which are effective for detecting high allele frequency differences between populations that have evolved over longer timescales, and haplotype-based selection scans that are designed to assess the direction of selection on more recent timescales (XP-EHH; Sabeti et al., 2007; Teshima et al., 2006). We found a higher proportion of selected loci in California residents when compared to the migrants, suggesting an adaptive suppression of migration in California. This same pattern was repeated in the east in comparisons between eastern residents and eastern migrants. This pattern is consistent with the hypothesis that residents represent derived forms that split off from ancestral migratory populations. In this context, the stronger selection signals in residents could reflect genetic changes associated with the suppression of migratory behavior, potentially allowing for the rapid shift from migratory to resident phenotypes while retaining the genetic basis of migration. Alternatively, we saw no difference in the proportion of selected loci between Arizona residents and migrants, supporting the idea that Arizona residents evolved independently from California residents. However, while our results are suggestive of a drop-off model, it is important to note that additional demographic analyses would be necessary to confirm this evolutionary scenario. Thus, while the observed directional selection in residents provides evidence about the adaptive suppression of migration, further work is needed to elucidate the demographic context of these findings.

Our analysis of the putative function of the candidate loci supported the idea that most of the selected loci were found in noncoding areas of the genome, suggesting that selection on putative regulatory regions, rather than coding genes, may underlie differences in migratory phenotypes. The importance of these cis-regulatory regions underlying phenotypic change has been more recently linked to the phenotypic differentiation within a radiation of capuchino seedeaters (Campagna et al., 2017). This result is in keeping with the idea that genetic variation at regulatory genes, rather than major changes to core mechanisms, may underlie the rapid modulation of migratory behavior within species.

### Parallel and convergent evolution

The frequency of parallel evolution is of interest to evolutionary biologists because it provides insight into the predictability and repeatability of the evolutionary process (Arendt & Reznick, 2008; Langerhans, 2018; Marques et al., 2022). In our study, we find strong signals of differentiation at 29,945 SNPs near or within 25 kb of 6,390 named genes across the genome, after adjusting for F_ST_ outliers linked to background population structure using comparisons within migrants and residents, but recognize that similar patterns can arise from neutral processes (Jensen et al., 2019). Of the total outlier loci, we identified 790 outlier SNPs across 42 genes with confirmed links to either migratory timing, the metabolic processes necessary for meeting the physiological demands of migratory flight, or morphology potentially important to the aerodynamics of flight. Of these, only one outlier SNP was shared among two comparisons, while 27 out of the 42 genes were shared among two or more comparisons (Figure 4). A similar pattern was seen in directional selection scans, where we identified putative directional selection signals at 64,794 SNPs near or within 25 kb of 4,005 named genes across the genome. Of the total selected loci, 972 SNPs were associated with 25 migration-linked genes. We found only 12 selected migration-linked loci were shared between resident populations, while 8 of 25 migration-linked genes were shared (Figure 4). Where the outlier loci are shared between different comparisons, we found that the majority of loci (9 of 11) have evolved in the same direction in resident populations compared to migratory populations. These results support the idea that parallel evolution is rare at the individual SNP level but may be more common at the genic level. The category with the largest number of genes containing outlier SNPs was migratory timing, followed by morphology, and then physiology. This may be because there are stronger signals of parallel evolution related to migratory timing than to the physiological or morphological aspects of flight or may be related to the fact that more is known about the genetic basis of migratory timing than the two other categories. Overall, our multi-pronged approach supports the existence of multiple possible evolutionary trajectories leading to the differentiation of migratory behavior, particularly at the individual SNP level. However, we also find support for parallel evolution at the genic level, particularly in genes linked to biological timekeeping.

### The genetics of migratory timing

Biological clocks are essential for coordinating and synchronizing physiological, behavioral, and metabolic processes related to migratory behavior in response to external environmental cues such as stress, temperature, and light (Cassone & Westneat, 2012; Kumar, 2017; Sharma et al., 2022). The core components of the clock gene network consist of positive elements (such as CLOCK, BMAL1, and NPAS) and negative elements (such as PER and CRY). The positive elements activate the expression of negative elements, which in turn inhibit their own expression. These core clock genes are further regulated by and, in turn, regulate genes within light input and metabolic sensor pathways located up and downstream of the clock gene network. Here we find that genes within light input and metabolic sensor pathways exhibited greater signals of parallel genetic changes when compared to the core clock genes. Specifically, we identified signals of parallel selection at two of the five core clock genes (*npas3* and *bmal*), with different sets being highlighted in different comparisons. In contrast, five light input or metabolic sensor pathway genes were specific to a single comparison (*phlpp1, peak1, creb1, rgs7,* and *top1*), while seven exhibited parallel changes in two or three comparisons (*gria2, camk4, ntrk2, hivep2, lgr4, ncor2*, and *rorb*). Further, all core clock genes, except *npas3*, were identified as important in other migratory bird comparisons (e.g., Delmore et al., 2015; Franchini et al., 2017), whereas only 11 out of the 18 circadian regulator genes were found in other migratory bird species (e.g., Jones et al., 2008; Singh et al., 2018). The observed lower frequency of parallel genetic changes in core clock genes versus clock regulator genes may be attributed to the higher conservation of these genes across species (Bazzi et al., 2016; Bossu et al., 2022; Le Clercq et al., 2023). As a result, there may be fewer options for genetic changes in core clock genes than in clock regulator genes, which might vary more by species.

We also identified signals of selection at five genes known to be controlled by the core clock network and two genes involved in the sleep-wake cycle. Notably, three of these genes were shared between all three comparisons and all were also identified as important in other migratory bird studies. While knockout studies have shown that clock-controlled genes are activated by the core CLOCK/BMAL1 complex (Hannou et al., 2018; Schroder et al., 2013; Takeuchi et al., 2023), the precise functions of these genes within the clock network are not known. However, the fact that three of them showed signals of parallel selection across all three of our comparisons suggests that they play an important role in differentiating migratory and resident forms of the Common Yellowthroat. Overall, the genes identified here as being important to two or more comparisons provide strong candidates for future research focused on testing the precise function of each gene in regulating migratory timing.

### Metabolism and energy expenditure

Metabolism plays a critical role in bird migration, enabling birds to undertake long-distance journeys and meet the energy demands associated with migration (Bossu et al., 2022; Kumar, 2017). Here we identified three genes with linkage to metabolism that showed signals of selection across all three of our comparisons. One of these genes, *st6galnac3*, is closely related to lipid metabolism (Altheide et al., 2006), which is the primary source of energy for long distance migrants. As part of their preparation in energy storage, most migrants increase their body mass significantly before migration and return to their normal condition at the end of it (Sharma et al., 2022). Even in species without season migration, cycles of gain and fat loss related to food availability throughout the annual cycle have been studied and linked to specific genes. The other gene, *fshr*, is known to be involved in fat deposition in Emu (*Dromaius novaehollandiae*: Wright et al., 2022) and was previously found as a regulator of abdominal adipose tissue of chickens (Cui et al., 2012). The third gene *tcf7l2* was differentially expressed in fast-growing and slow-growing chickens with differences in fat deposition (Claire D’Andre et al., 2013). Energy storage as fat has also related to hormones that control lipid metabolism (Wensveen et al., 2015). We also identified two genes (*pdxk,* and *sorbs1*) with links to metabolic processes, which were shared across at least two of our comparisons. Of these genes, *pdxk* is known to be involved in the formation of adipocytes, which are specialized cells whose primary function is to store energy in the form of fat. Parallel signals of evolutionary change at the level of genes linked to fat storage and synthesis highlight the important role of these traits in meeting the energy demands of long-distance flight.

### Morphology potentially related to the aerodynamics of flight

Birds have evolved several morphological adaptations that aid in their migration, enabling them to undertake long-distance journeys. These adaptations are generally geared towards enhancing flight efficiency, endurance, and navigation abilities (Phillips et al., 2018; Vágási et al., 2016). Here we identify six genes (*acvr2b, tnfrsf11a, ablim1, palld, pkdcc*, and *ppp3ca*) with links to muscle mass, bone mass, and muscle development, of which five are shared across at least two comparisons and two of which are shared across all three comparisons. Further, all but one of these (*tnfrsf11a*) was found in other avian studies. While direct linkages with migration behavior are less obvious for the morphology linked genes identified here than for those linked to migration timing and physiology, bone and muscle mass are critical for maintaining the proper weight to lift ratio necessary for long-distance flight (Louis et al., 2022; Vágási et al., 2016). Further factors, such as wing morphology, muscle strength, and respiratory capacity, also contribute significantly to flight performance and are known to vary between populations with differing levels of migratory behavior. This work serves as a start towards documenting genes with potential links to the aerodynamics of flight, which are coming out as important across multiple avian species.

## Conclusions

In this study, we employed a comparative genomic approach to gain valuable insights into the predictability and repeatability of the genes underlying migratory behavior. By analyzing genome-wide genetic data collected using the outlier and selection scans across replicated populations differing in migratory phenotypes, we identify suites of genes with parallel signals of genetic change as well as multiple examples of genetic convergence. While further work is necessary, we found the strong signals of genetic parallelism in genes linked to light input and metabolic sensor pathways, the core clock network, the flight energetics and morphology potentially related to flight aerodynamics. Alternatively, genes upstream or downstream of the core clock network were less similar across population and species level comparisons. Our study provides compelling evidence that the evolution of complex phenotypes, such as migratory behavior, is not constrained to a single pathway. Instead, our findings demonstrate the existence of multiple possible evolutionary trajectories leading to the shifts of this behavior. This work highlights the potential flexibility and adaptability of organisms in responding to environmental challenges in a changing world.

## Supplementary material

Supplementary material is available online at *Evolution Letters*.

qrae064_suppl_Supplementary_Material

## Data Availability

Individual genotype data, including called genotypes or genotype probability from low coverage whole genome sequence data, corresponding metadata files and custom scripts are available on Dryad: https://doi.org/10.5061/dryad.4j0zpc8j9
