# Supplementary material for: Parallel and convergent evolution in genes underlying seasonal migration
Source: Evol Lett. 2024 Nov 30;9(2):189–208. doi: 10.1093/evlett/qrae064 (PMC11968193; doi:10.1093/evlett/qrae064)
Supplement: qrae064_suppl_Supplementary_Material [file qrae064_suppl_supplementary_material.docx]

**Supplementary Results.**

**F_ST_ outlier scan**

Here, we combined F_ST_ outlier scans and two selection scans to identify candidate loci underlying differentiation in 3 replicate resident-migrant comparisons. F_ST_ outlier scans showed distinct patterns between the West and East. Specifically, overall background genetic differentiation between replicate migratory-resident comparisons in the West was higher, with a total weighted average F_ST_ of 0.0202 and 0.0339 for AZ and northern CA vs. western migrants (Figure 2A, B), respectively. The mean F_ST_ among the 50kb sliding windows was similarly high (mean F_ST_AZ_=0.0216, 99 CI_AZ_: 0.0214- 0.0218, mean F_ST_CA_= 0.0374, 99 CI_CA_: 0.0371- 0.0377). The elevated background genomic differentiation was not found in the West migrant-migrant comparison, i.e. the global weighted F_ST_ was -0.0085 (Figure S3A). In contrast, overall genetic differentiation between the migratory-resident populations in the East was much lower with a total weighted average F_ST_ of 0.0141 among biallelic SNPs and a mean F_ST_ among sliding 50 kb windows of 0.0184 (99 CI_East_: 0.0178- 0.0189).

We identified a total of 22,808 and 14,249 outlier loci in the AZ vs West and CA vs. West migrant comparisons, respectively, after accounting for outlier loci identified in the West migrant-migrant and resident-resident comparisons. In the Arizona comparison, 5,507 were in or near genic regions (within 25 kb) and only 114 variants were associated with 29 migration-linked genes. Similarly, in the California comparison, 3,290 loci were found in genic regions, but only 70 outlier loci were associated with 21 candidate migration-linked genes after removing western migrant-migrant and resident-resident outlier variants. Six genes were shared between the Arizona and California resident-migrant comparisons. In the East comparison, we identified 49,684 outlier loci, with 21,260 in or around genic regions. Of those, 608 loci were associated with 28 migration-link genes and not identified in the east migrant-migrant comparison. Eight genes were shared between the AZ and the East resident-migrant comparisons, and four genes were shared between CA and East resident-migrant comparisons, and nine genes were shared between all three resident-migrant comparisons (Table 1; Figure 4).

**Genome-wide selection scan**

We identified clear signals of putative selection in each replicate resident-migration comparison using genome-wide selection scans in the East and West, separately (Figure S4). In the resident vs migrant angsd selection scans, we identified 239,703 selected loci (p-value = 0.05) in the East resident vs East migrant comparison (Figure S4 A, C; PC1), 164,324 selected loci associated with the AZ resident vs. West migrant comparison (Figure S4 B, C; PC2) and 154,172 selected loci associated with the northern CA vs. West migrant comparison (Figure S4, D; PC1). In the East selection scan 50,424 of the selected loci fell within 25kb of 7,917 named genes. Of those variants, 1,167 fell near 41 migration-linked genes. In the AZ selection scan, 37,134 of selected loci were found within 25kb of 7422 named genes. Of those, 447 loci were found in 40 migration-linked genes. In the northern California selection scan, 34,031 selected loci were found within 25kb of 7,425 named genes, with 367 loci linked to 42 migration-linked genes.

We extended our analyses to include directional selection scans to pinpoint on which population selection was acting in the 3 replicate comparisons. We identified 43,246

selected loci in the East resident vs. East migrant comparison, 20,430 loci that were in or near 2,412 genes. Of those, 13939 are linked to selection in 1947 genes in the East resident population. In the northern California comparison, we identified 41,736 loci in selective sweeps, 22,305 in or near 1,654 genes. Similar to the east, the majority of sites (20,554) and genes (1,485), were linked to selective sweeps in the resident population. A similar number of selected loci were identified in the Arizona resident comparison, 42,369 loci, 25,283 in or near 1,783 genes. However, the skewed selection signal in the resident population was not the pattern seen. Only 11,410 loci were selected in the AZ population and 735 genes showed signals of selective sweep in AZ, whereas 1,048 genes showed a selective sweep in the West migrant population.

**Supplementary Figures and Table.**

**Figure S1**. Cross validation (CV) error plots used for K selection in geographic structure analysis (AdmixPipe), for West and East main groups (A). Admixture plots for each K value evaluated from K=2 through K=6 (B).


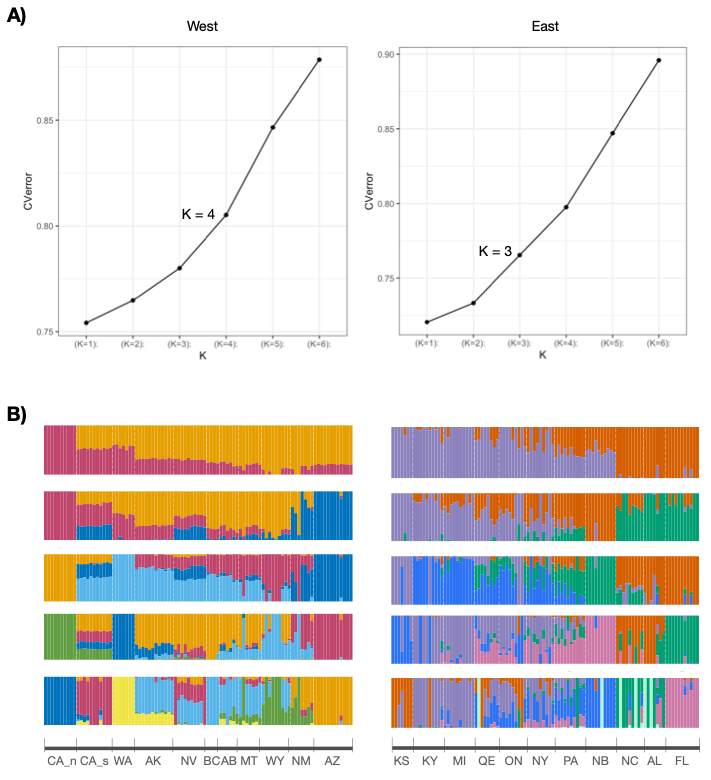


**Figure S2.** Principal Component Analysis plots used to assess genomic variation and geographic structure. PCA plot for the whole complex (A). PCA plot for the Eastern group (B). PCA plot for the Western group. Different color groups match to the main clusters found in Admixture results (see Fig. 1).


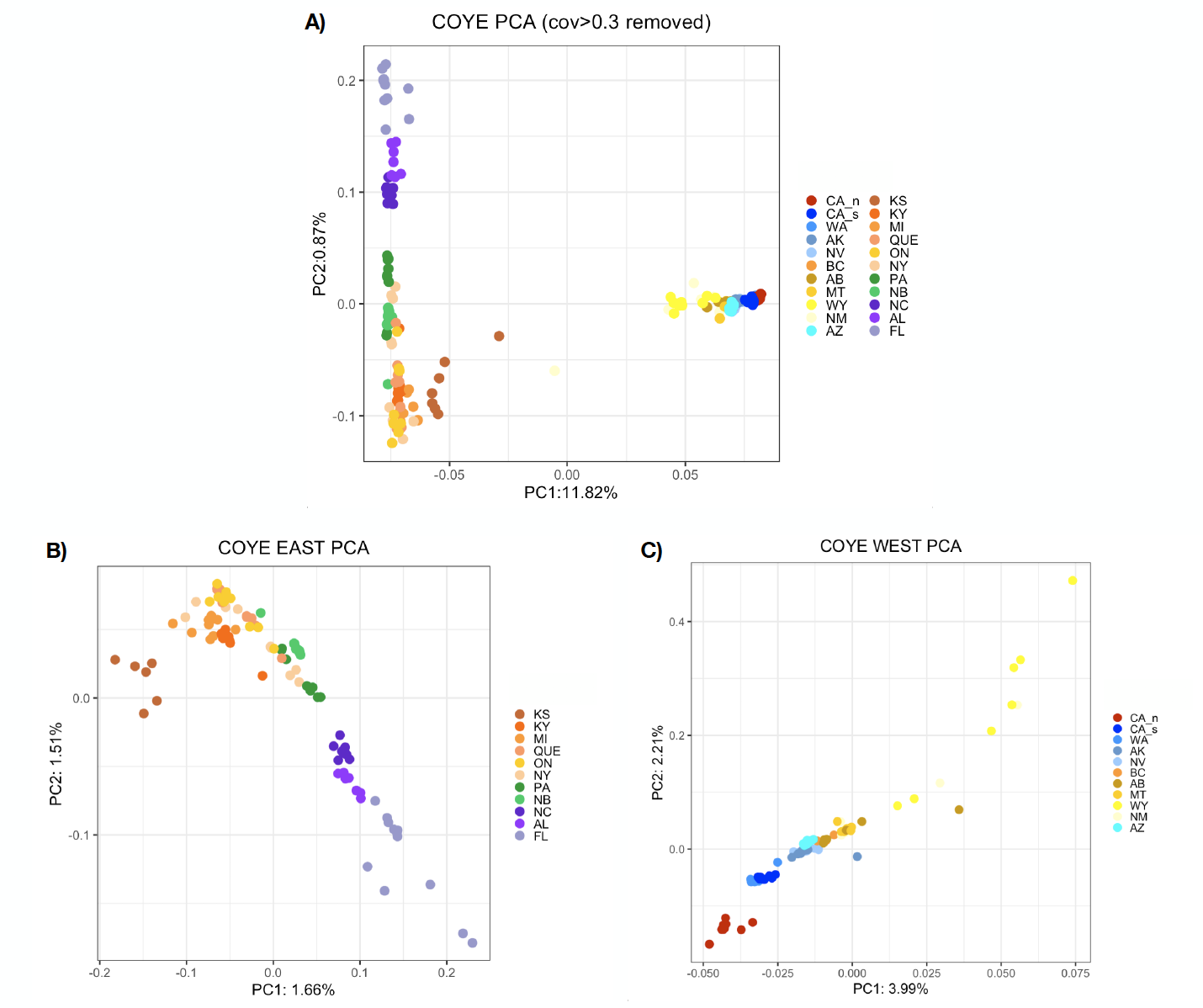


**Figure S3.** Manhattan plots showing F_ST_ estimates of genomic differentiation patterns of 50kb sliding windows across the genome. Migratory paired populations were used to visualize outliers in A) Western and B) Eastern migrants respectively, thus not linked to the migratory phenotype.


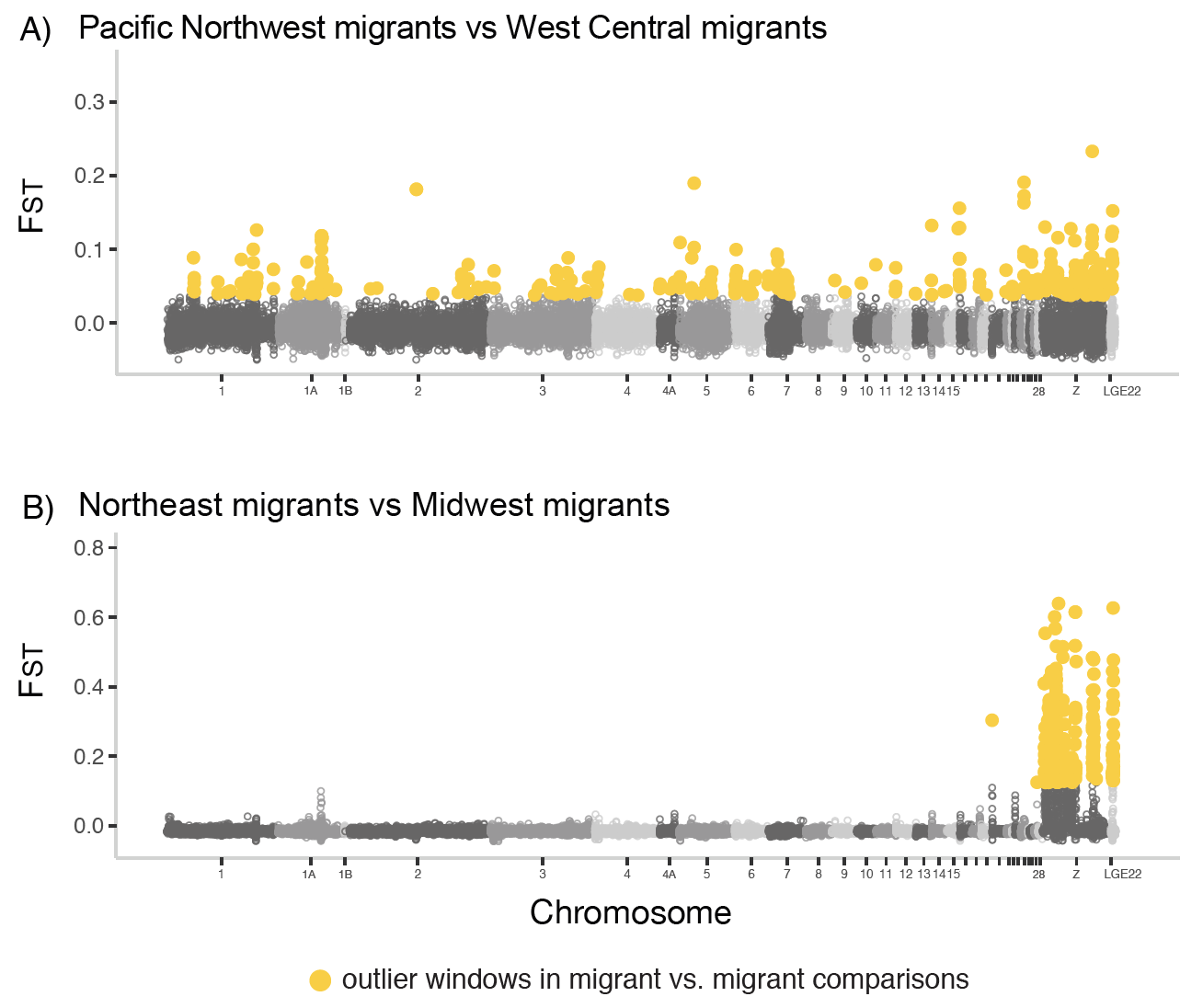


**Figure S4.** Selection scan in PCANGSD of three replicate comparisons. The PCA of A) East and B) West separately that show East resident populations, FL and AL, are differentiated on PC1, while CA resident cluster on PC1 (red) and AZ resident cluster on PC2 (cyan). Manhattan plots of selected variants (p-value < 0.05) are highlighted in yellow for C) Easter residents vs. Eastern migrant, D) AZ residents vs. Western migrants, and E) northern CA residents vs. Western migrants.


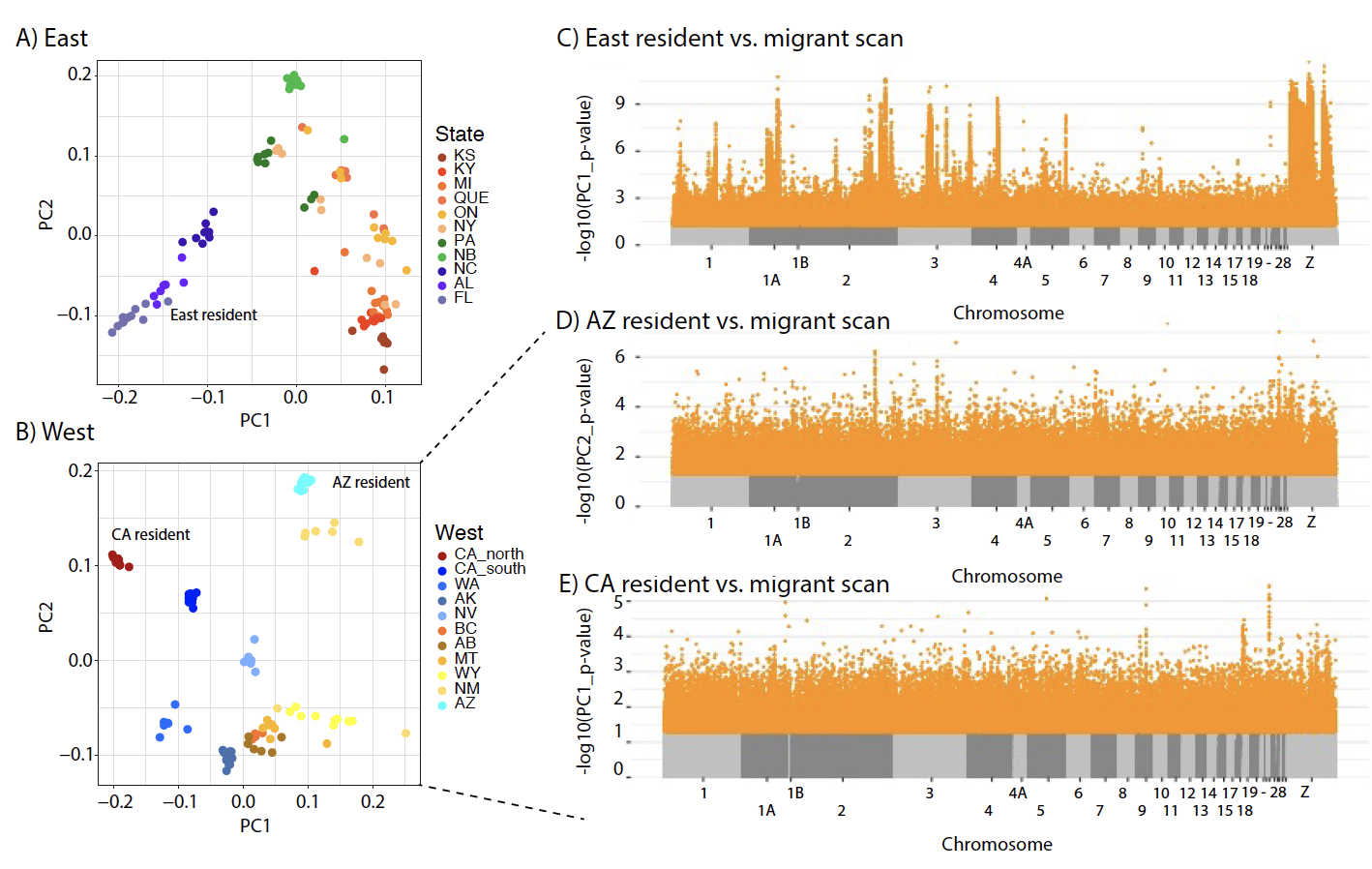


**Table S1**. Samples of the Common yellowthroat used in this study (*Geothlypis trichas*). Information about individual IDs, Locality information, including map code (see Figure 1), and migratory phenotype is included.

| **Map code** | **Sample ID** | **Country** | **State** | **Phenotype** | **Region** | **Lat** | **Long** |
| --- | --- | --- | --- | --- | --- | --- | --- |
| 1 | 17N00997, 17N01005,  17N01007, 17N01013,  17N01014, 17N01018,  17N01020, 17N02629,  17N02630, 17N02631,  17N02632, 17N02642 | USA | AK | Migrant | West | 56.59 | -132.76 |
| 2 | 03N2087, 03N2090,  03N2107, 03N2110 | Canada | BC | Migrant | West | 50.90 | -118.48 |
| 3 | 17N02809, 17N02810  17N02828, 17N02829,  17N02831, 2730-70640 | Canada | AB | Migrant | West | 52.91 | -118.11 |
| 4 | 02N8390, 02N8391,  02N8392, 02N8420,  02N8421, 02N8423,  02N8424, 02N8425 | USA | WA | Migrant | West | 47.03 | -122.34 |
| 5 | 17N02619, 17N02620,  17N02621, 17N02622,  17N02623, 17N02625,  17N02626, 2780-13031 | USA | MT | Migrant | West | 47.22 | -113.53 |
| 6 | 17N02811, 17N02816,  17N02817, 17N02819,  17N02820, 17N02826,  99N0579, 99N0580,  99N0581 | USA | WY | Migrant | West | 44.38 | -104.77 |
| 7 | 02N8885, 02N8886,  02N8887, 02N8889,  02N8893, 02N8895,  02N8901, 02N8931,  03N6158, 03N6159 | USA | NV | Migrant | West | 40.2 | -115.48 |
| 8 | 16N0001, 16N0005,  16N0006, 16N0007,  16N0008, 16N0009,  16N0010, 16N0011,  16N0012, 16N0013,  16N0014, 16N4313 | USA | CA_n | Resident | West | 38.17 | -122.90 |
| 9 | 16N4301, 16N4302,  16N4303, 16N4305,  16N4307, 16N4312,  17N02653, 17N02661,  17N02662, 17N02681,  17N02822 | USA | CA_s | Migrant | West | 33.27 | -117.37 |
| 10 | 09N15821, 10N17234,  10N17235, 10N17236,  11N0358, 11N0359,  11N0363, 11N0364 | USA | NM | Migrant | West | 33.48 | -104.42 |
| 11 | AZCOYE15, AZCOYE16,  AZCOYE18, AZCOYE19,  AZCOYE20, AZCOYE21,  AZCOYE22, AZCOYE23,  AZCOYE24, AZCOYE27,  AZCOYE28, AZCOYE29 | USA | AZ | Resident | West | 31.46 | -110.26 |
| 12 | 98N2035, 98N2037,  98N2039, 98N2042,  98N2047, 98N2051,  98N2055, 98N2058 | Canada | QE | Migrant | East | 48.83 | -72.54 |
| 13 | 98N2061, 98N2064,  98N2065, 98N2072,  98N2073, 98N2078,  98N2086, 98N2089,  98N2095, 98N2096 | Canada | ON | Migrant | East | 47.74 | -79.70 |
| 14 | 98N2006, 98N2008,  98N2009, 98N2012,  98N2013, 98N2016,  98N2017, 98N2018,  98N2021, 98N2022 | Canada | NB | Migrant | East | 45.93 | -66.32 |
| 15 | 16N0238, 16N0239,  16N0241, 16N0242,  16N0243, 16N0246,  16N0249, 16N0261,  16N4261, 2700-15676 | USA | MI | Migrant | East | 42.30 | -85.32 |
| 16 | 16N4237, 16N4239,  16N4241, 16N4248,  16N4250, 16N4251,  16N4253, 16N4254,  16N4255 | USA | NY | Migrant | East | 43.32 | -77.72 |
| 17 | 01N4486, 01N4491,  01N4492, 01N4493,  02N1424, 02N1427,  02N1428 | USA | KS | Migrant | East | 39.02 | -96.84 |
| 18 | 16N1052, 16N1065,  16N1081, 16N4287,  16N4290, 16N4291,  16N4292, 16N4293,  16N4294, 17N02605 | USA | KY | Migrant | East | 37.81 | -84.76 |
| 19 | 16N4334, 16N4336,  16N4337, 16N4340,  16N4341, 16N4342,  16N4346, 16N4347,  16N4348, 16N4353 | USA | PA | Migrant | East | 40.66 | -75.67 |
| 20 | 04N9069, 04N9070,  09N0194, 99N7114,  99N7115, 99N7117,  99N7118, 99N7119,  99N7120 | USA | NC | Migrant | East | 35.12 | -79.32 |
| 21 | 09N20305, 14N0311,  14N0313, 16N0354,  16N0356, 16N0357,  16N0358 | USA | AL | Resident | East | 32.04 | -85.47 |
| 22 | BAY_07, BRY_12,  GOM_07, MOR_12,  MRG_07, NAB_07,  NGO_12, OBO_12,  OBY_07, RAW_07,  WNM_07, WYM_07 | USA | FL | Resident | East | 27.22 | -81.38 |

AB: Alberta, AK: Alaska, AL: Alabama, AZ: Arizona, BC: British Columbia, CA: California, CA_n: north California, CA_s: south California, FL: Florida, KS: Kansas, KY: Kentucky, MI: Michigan, MT: Montana, NB: New Brunswick, NC: North Carolina, NM: New Mexico, NV: Nevada, NY: New York, ON: Ontario, PA: Pennsylvania, QE: Quebec, WA: Washington, WY: Wyoming.

**Table S2**. Number and proportion of loci found in directional selection scans (XP-EHH).

| Comparison | Populations | Total loci | Selected migratory loci: resident | Selected migratory loci: migrant | Selected migratory genes: resident | Selected migratory genes: migrant | Intergenic loci | Synonymous loci | Intron region loci | Upstream and downstream region loci | Missense loci |
| --- | --- | --- | --- | --- | --- | --- | --- | --- | --- | --- | --- |
| A | Arizona vs. West migrants | 420 | 181 (43.1%) | 239 (56.9%) | 7 | 13 | 97 | 0 | 206 | 117 (10 genes) | 0 |
| B | northern California vs West migrants | 339 | 314 (92.6%) | 25 (7.4%) | 16 | 2 | 6 | 4 | 300 | 28 (7 genes) | 1 |
| C | Florida and Alabama vs East migrants | 748 | 564 (75.4%) | 184 (24.6%) | 13 | 7 | 277 | 2 | 426 | 43 (8 genes) | 0 |
